# Supplementary material for: Biological and Genetic Characterizations of a Novel Lytic ΦFifi106 against Indigenous Erwinia amylovora and Evaluation of the Control of Fire Blight in Apple Plants
Source: Biology (Basel). 2023 Jul 28;12(8):1060. doi: 10.3390/biology12081060 (PMC10452218; doi:10.3390/biology12081060)
Supplement: Supplementary file 1 [file biology-12-01060-s001.zip › biology-2482745-supplementary.pdf]

Table S1. Characteristics of *E. amylovora*-specific myophages reported in scientific papers (2009 to the present).

Phage	Morphology		Genome				One-step		Accession number	Reference
	Head length (nm)	Tail length (nm)	Genome size (bp)	GC (%)	ORF	tRNA	Latent period (min)	Burst size (PFU/cell)		
**ΦFifi106**	**80**	**114**	**84,405**	**43.4**	**114**	**26**	**20**	**310**	OR284297	**This study**
pEa_SNUABM_27	69	115	53,014	44.1	78	1	–[1)]	–	MW349138.1	[1]
pEa_SNUABM_31	139	196	265,846	49.5	337	0	–	–	MZ443773.1	[1]
pEa_SNUABM_32	130	169	265,891	49.2	336	0	–	–	MZ443774.1	[1]
pEa_SNUABM_48	140	150	294405	49.5	358	2	–	–	MW879340.1	[1]
pEa_SNUABM_12	130	127	358,115	34.4	546	32	40	18	MT939486.1	[2]
pEa_SNUABM_47	128	127	355,376	34.5	540	35	40	20	MT939487.1	[2]
pEa_SNUABM_50	130	127	356,948	34.5	540	34	40	16	MT939488.1	[2]
Hena1	72	126	148 842	48.4	240	23	–	–	MN732867.1	[3]
vB_EamM_Bosolaphorus	128	159	272,228	49.4	321	1	–	–	MG655267.1	[4]
vB_EamM_Desertfox	128	159	272,458	49.6	320	0	–	–	MG655268.1	[4]
vB_EamM_MadMel	128	159	275,000	49.4	321	0	–	–	MG655269.1	[4]
vB_EamM_Mortimer	128	159	273,914	49.5	325	1	–	–	MG655270.1	[4]
vB_EamM_RAY	128	159	271,182	49.9	319	1	–	–	KU886224.1	[4]
vB_EamM_Simmy50	128	159	271,088	49.9	322	1	–	–	KU886223.1	[4]
vB_EamM_Special G	128	159	273,224	49.8	324	0	–	–	KU886222.1	[4]
vB_EamM_Deimos-Minion	128	159	273,501	49.9	326	0	180–240	5	KU886225.1	[4]
vB_EamM_RisingSun	143	207	235,108	48.3	243	0	–	–	MF459646.1	[5]
vB_EamM_Joad	143	207	235,374	48.3	245	0	–	–	MF459647.1	[5]
vB_EamM_Y3	129	192	261,365	47.0	333	0	–	–	KY984068.1	[6]
vB_EamM-Bue1	79	126	164,037	50.2	175	1	–	–	MG973030.1	[7]
vB_EamM-Y2	67	124	56,621	44.2	90	–	–	–	HQ728264.1	[8]
vB_EamM-M7	77	116	84,694	43.4	117	–	–	–	HQ728263.1	[8]
phiEa21-4	60	90	84,576	43.8	117	26	–	–	EU710883.1	[8]
phiEa104	72	114	84,565	43.9	118	24	–	–	FQ482083.1	[9]

[1)] –, not provided.

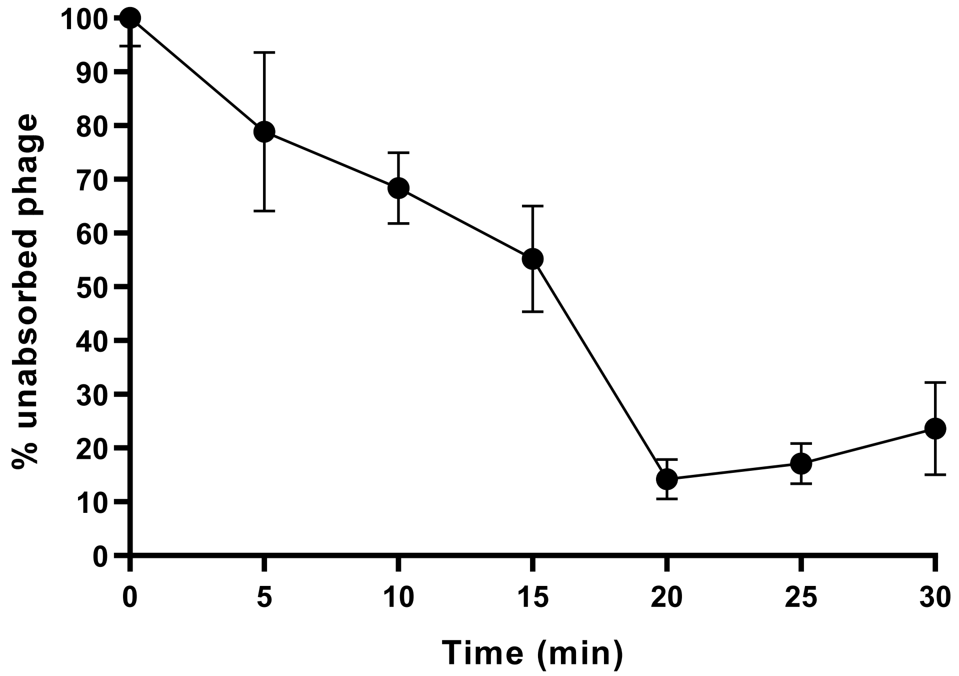

Figure S1. Adsorption assay of ΦFifi106.

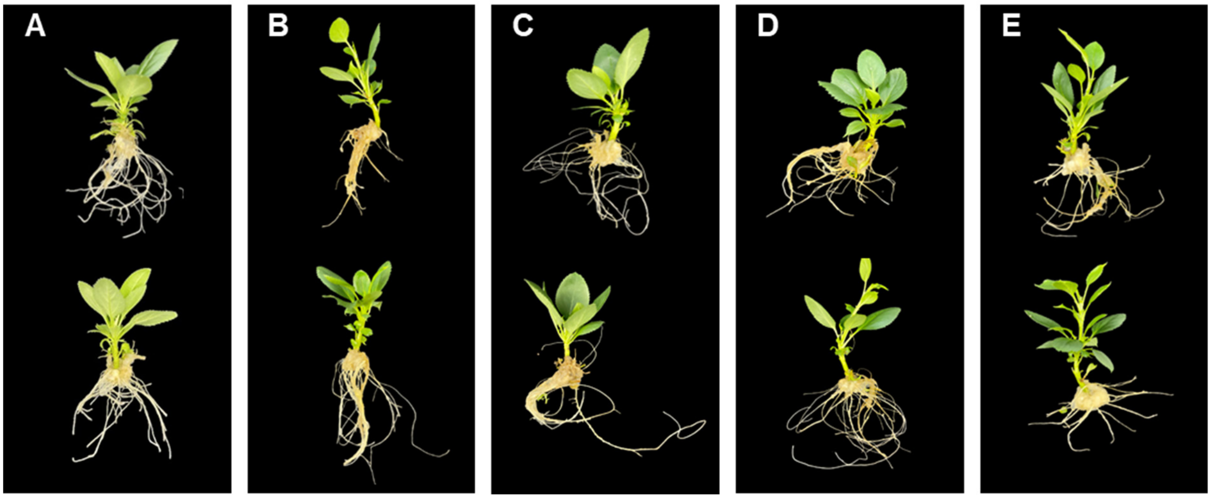

Figure S2. The representative images of M9 apple plants (A) before the treatment, and after the treatment of (B) sterilized water, (C) ΦFifi106, (D) AgriPhage-FireBlight, and (E) Bramycin, respectively.

References

1. Kim, S.-G.; Lee, S.-B.; Jo, S.-J.; Cho, K.; Park, J.-K.; Kwon, J.; Giri, S.S.; Kim, S.-W.; Kang, J.-W.; Jung, W.-J. Phage cocktail in combination with kasugamycin as a potential treatment for fire blight caused by *Erwinia amylovora*. *Antibiotics* **2022**, *11*, 1566.
2. Kim, S.G.; Lee, S.B.; Giri, S.S.; Kim, H.J.; Kim, S.W.; Kwon, J.; Park, J.; Roh, E.; Park, S.C. Characterization of novel *Erwinia amylovora* jumbo bacteriophages from Eneladusvirus genus. *Viruses* **2020**, *12*, 1373.
3. Besarab, N.V.; Akhremchuk, A.E.; Zlatohurska, M.A.; Romaniuk, L.V.; Valentovich, L.N.; Tovkach, F.I.; Lagonenko, A.L.; Evtushenkov, A.N. Isolation and characterization of Hena1–a novel *Erwinia amylovora* bacteriophage. *FEMS Microbiol. Lett.* **2020**, *367*, fnaa070.
4. Sharma, R.; Pielstick, B.A.; Bell, K.A.; Nieman, T.B.; Stubbs, O.A.; Yeates, E.L.; Baltrus, D.A.; Grose, J.H. A novel, highly related jumbo family of bacteriophages that were isolated against Erwinia. *Front. Microbiol.* **2019**, *10*, 1533.
5. Arens, D.K.; Brady, T.S.; Carter, J.L.; Pape, J.A.; Robinson, D.M.; Russell, K.A.; Staley, L.A.; Stettler, J.M.; Tateoka, O.B.; Townsend, M.H. Characterization of two related *Erwinia* myoviruses that are distant relatives of the PhiKZ-like Jumbo phages. *PloS one* **2018**, *13*, e0200202.
6. Buttimer, C.; Born, Y.; Lucid, A.; Loessner, M.J.; Fieseler, L.; Coffey, A. *Erwinia amylovora* phage vB_EamM_Y3 represents another lineage of hairy *Myoviridae*. *Res. Microbiol.* **2018**, *169*, 505-514.
7. Knecht, L.E.; Born, Y.; Pothier, J.F.; Loessner, M.J.; Fieseler, L. Complete genome sequences of *Erwinia amylovora* phages vB_EamP-S2 and vB_EamM-Bue1. *Microbiol. Resour. Ann.* **2018**, *7*, e00891-00818.
8. Born, Y.; Fieseler, L.; Marazzi, J.; Lurz, R.; Duffy, B.; Loessner, M.J. Novel virulent and broad-host-range *Erwinia amylovora* bacteriophages reveal a high degree of mosaicism and a relationship to Enterobacteriaceae phages. *Appl. Environ. Microbiol.* **2011**, *77*, 5945-5954.
9. Müller, I.; Lurz, R.; Kube, M.; Quedenau, C.; Jelkmann, W.; Geider, K. Molecular and physiological properties of bacteriophages from North America and Germany affecting the fire blight pathogen *Erwinia amylovora*. *Microb. Biotechnol.* **2011**, *4*, 735-745.
